# P-1365. Experience of Infliximab Use for Patients with Central Nervous System Tuberculosis in North West London

**DOI:** 10.1093/ofid/ofaf695.1552

**Published:** 2026-01-11

**Authors:** Anna Daunt, Alastair McGregor, Cassandra Watson, Denis Keane, Ashley Whittington, Christopher Dugan, Gabriel Wallis, Tumena Corrah, Laurence John, Padmasayee Papineni

**Affiliations:** London North West University Healthcare NHS Trust, London, England, United Kingdom; London North West University Healthcare NHS Trust, London, England, United Kingdom; London North West University Healthcare NHS Trust, London, England, United Kingdom; London North West University Healthcare NHS Trust, London, England, United Kingdom; London North West University Healthcare NHS Trust, London, England, United Kingdom; London North West University Healthcare NHS Trust, London, England, United Kingdom; London North West University Healthcare NHS Trust, London, England, United Kingdom; London North West University Healthcare NHS Trust, London, England, United Kingdom; London North West University Healthcare NHS Trust, London, England, United Kingdom; London North West University Healthcare NHS Trust, London, England, United Kingdom

## Abstract

**Background:**

Central nervous system tuberculosis (CNS TB) has high mortality and morbidity and can be further complicated by paradoxical reactions. There is increasing use of infliximab for steroid-resistant inflammation in CNS TB. We reviewed data from patients with CNS TB treated with infliximab in a diverse area of London with one of the highest TB endemicity in the UK.

**Table 1:**
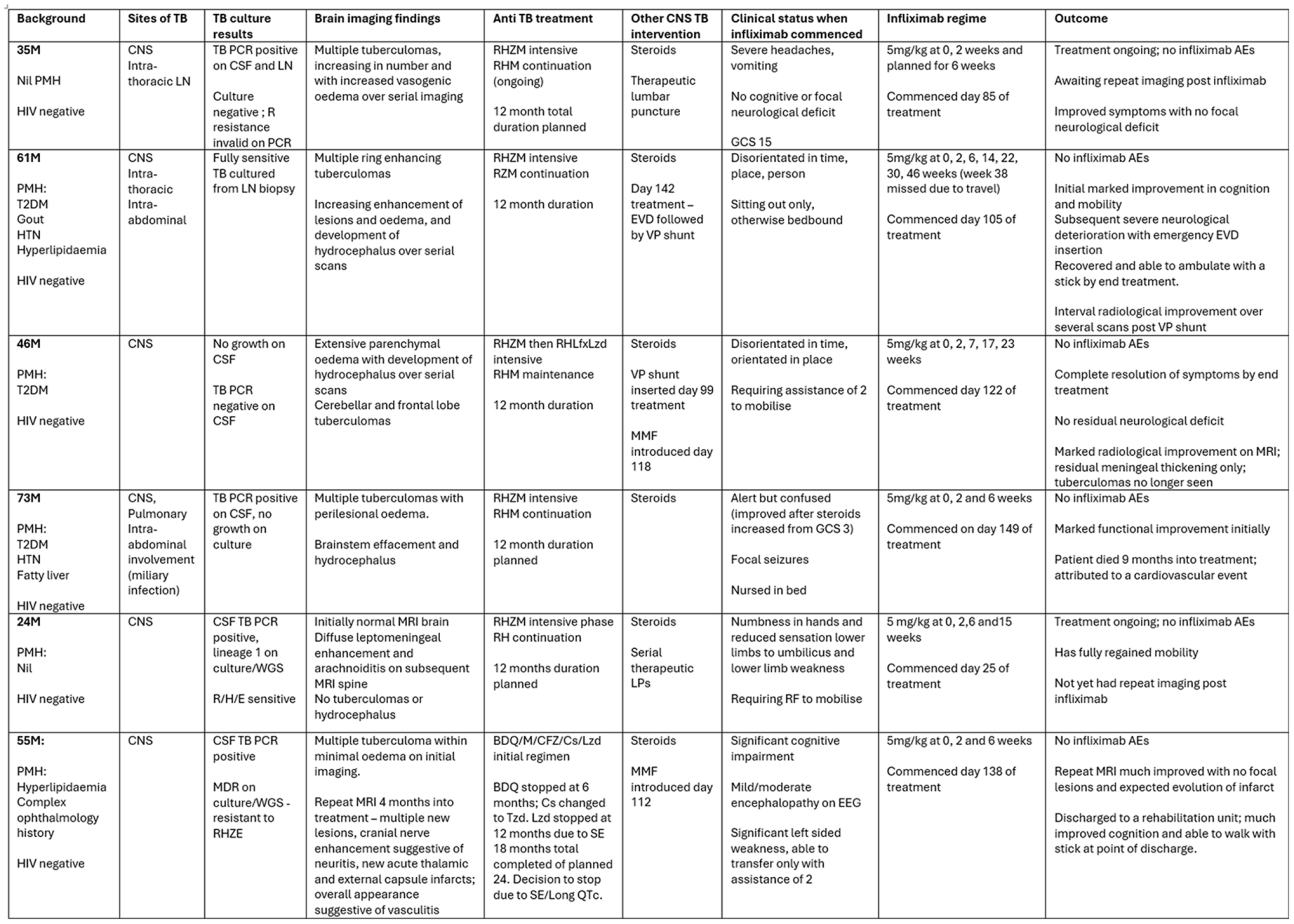
Clinical information, radiological findings, TB treatment regimen and outcomes Abbreviations: AE; adverse events; BDQ; bedaquiline; CFZ; clofazamine; CNS; central nervous system; Cs; cycloserine; CSF; cerebrospinal fluid; E; ethambutol; EEG; electroencephalogram; EVD; external ventricular drain; GCS; Glasgow Coma Scale; H; isoniazid; HIV; human immunodeficiency virus; HTN; hypertension; Lfx; levofloxacin; LN; lymph node; LP; lumbar puncture Lzd; linezolid; M; moxifloxacin; MMF: mycophenolate mofetil MRI; magnetic resonance imaging; PCR; polymerase chain reaction; PMH; past medical history; QTc: corrected QT interval R; rifampicin; RF; rollator frame; SE side effects; VP; ventriculoperitoneal; T2DM; Type 2 Diabetes Mellitus; TB; tuberculosis; Tzd; terizidone WGS; whole genome sequencing; Z; pyrazinamide

**Methods:**

Retrospective analysis of medical records, radiology and microbiology results of patients with CNS TB identified from the London TB register and pharmacy records from 2022 from London North West Hospitals University NHS Trust.

**Results:**

Since 2022, 1134 patients were treated for TB and 93 for CNS TB. 6 patients with CNS TB received infliximab of whom all were male; aged 24-73 years. 3 patients had culture confirmed TB, 2 had positive TB PCR and 1 was treated empirically. 5 patients were treated for drug sensitive TB and 1 for multi-drug resistant TB. Comorbidities, full regimes, and neuroimaging findings are detailed in Table 1. All received steroids using Thwaites protocol; 2 also received mycophenolate mofetil. 2 patients received therapeutic lumbar punctures, and 2 required neurosurgical intervention for hydrocephalus.

All patients were treated with infliximab following clinical and radiological deterioration despite treatment with anti-TB medications and steroids. 5mg/kg was given to each patient, commenced between 25 and 149 days after TB treatment started. All patients received or are planned to receive doses at 0, 2 and 6 weeks. 3 patients received a further 1-4 doses at 6-8 week intervals. Following infliximab 4 patients had improvement in symptoms, cognition and/or functional status. Of these, 2 had radiological improvement; 2 are awaiting repeat imaging. 1 patient initially improved then had a severe deterioration requiring neurosurgery and has now recovered to a better functional status than prior to infliximab therapy. 1 patient died 9 months into TB treatment, attributed to a likely cardiovascular event.

**Conclusion:**

Our experience supports previous studies suggesting infliximab may be a useful treatment for paradoxical reaction and severe inflammation in CNS TB. Randomised controlled trials are now needed to definitively determine efficacy, dosing and timing.

**Disclosures:**

All Authors: No reported disclosures

